# Determinants of hospital length of stay in ischemic stroke patients: A retrospective cohort study at Indonesian national stroke center

**DOI:** 10.1371/journal.pone.0334355

**Published:** 2025-11-14

**Authors:** Mursyid Bustami, Sri Idaiani, Nadira Deanda Putri, Ari Winasti Satyagraha, Novaria Sari Dewi Panjaitan, Anna Mardiana Ritonga, Anyelir Nielya Mutiara Putri, Shofiya Rohmah Asyahida, Mei Sarah Nurkhalizah, Diva Azizah Nitisara, Beny Rilianto, Nugroho Harry Susanto, Yuli Felistia, Anwar Santoso

**Affiliations:** 1 Clinical Research Unit, National Brain Center Hospital Mahar Mardjono Jakarta, Jakarta, Indonesia; 2 Department of Neurology, National Brain Center Hospital Mahar Mardjono Jakarta, Jakarta, Indonesia; 3 Research Center for Preclinical and Clinical Medicine, National Research and Innovation Agency, Bogor, Jawa Barat, Indonesia; 4 Center for Biomedical Research, National Research and Innovation Agency, Bogor, Jawa Barat, Indonesia; 5 Eijkman Research Center for Molecular Biology, National Research and Innovation Agency, Bogor, Jawa Barat, Indonesia; 6 Department of Cardiology - Vascular Medicine, National Cardiovascular Center – Harapan Kita Hospital, Jakarta, Indonesia; STIKES Wira Medika PPNI Bali: Sekolah Tinggi Ilmu Kesehatan Wira Medika PPNI Bali, INDONESIA

## Abstract

**Background:**

Stroke is a major cause of death and disability, with prolonged hospitalization driving up healthcare costs. This study investigated factors influencing length of stay (LOS) in ischemic stroke patients at a leading Indonesian stroke center.

**Methods:**

A retrospective cohort study was conducted on 2,804 ischemic stroke patients admitted in 2020. Univariable and multivariable zero truncated negative binomial regression analyses were performed using R Statistical Software (v4.5.1) to identify factors significantly associated with LOS.

**Results:**

The study population had a mean age of 61.3 years (SD ± 11.4), with a predominance of male patients (63.5%). The average length of LOS was 5.1 days (SD ± 3.4). Several factors were significantly associated with LOS, including ward class (hospital ward class II: 0.91 IRR, 95% CI:0.86–0.97; VIP class: 0.87 IRR, 95%CI:0.79–0.96), payment method (private/co-share: 1.13 IRR, 95%CI:1.03–1.25; out-of-pocket: 1.19 IRR, 95%CI:1.09–1.30), and stroke severity (moderate: 1.16 IRR, 95%CI:1.11–1.21; severe: 1.56 IRR, 95%CI:1.44–1.69). Additionally, elevated blood pressure (0.85 IRR, 95%CI:0.73–0.98), stage 1 hypertension (0.90 IRR, 95%CI:0.82–0.99), diabetes mellitus (1.09 IRR, 95%CI:1.04–1.15), dyslipidemia (0.95 IRR, 95%CI:0.90–0.999), all at admission, and cardiovascular disease (CVD) history (1.24 IRR, 95%CI:1.19–1.29), hospital complications (1.99 IRR, 95%CI:1.87–2.12), and therapeutic interventions (1.38 IRR, 95%CI:1.29–1.48) were also significantly linked to LOS. A subgroup analysis showed that CVD history, hospital complications, and therapeutic interventions during hospitalization were significantly linked to the LOS across all levels of stroke severity.

**Conclusion:**

CVD history, hospital complications, and therapeutic interventions significantly influenced LOS across stroke severities. Early intervention, complication prevention, and equitable care are essential to shorten hospitalization, reduce costs, and improve outcomes in Indonesian stroke patients.

## Introduction

Stroke is one of the leading causes of death and disability worldwide [1]. The Global Burden of Disease Study, spanning from 1990 to 2019, reported an increase in stroke-related morbidity and mortality globally [2]. Besides its health impact, stroke also leads to significant financial burden, with estimated global healthcare costs exceeding US$891 billion each year [3]. In 2023, the Social Security Agency on Health reported spending IDR 5.2 trillion (around USD 318 million) on stroke care, ranking it among the top three diseases with the highest healthcare expenditures in Indonesia [4]. The length of hospital stay (LOS) represents a critical determinant of healthcare expenditure, as it directly impacts the overall cost of treatment and thus intensifies the economic burden associated with stroke [5].

LOS is defined as the total number of days a patient spends in the hospital, starting from admission in the emergency department until discharge from the ward. It serves as a key indicator of resource utilization within a hospital. In general, a longer LOS corresponds to higher hospitalization costs. This correlation between LOS and treatment expenses poses a major challenge for healthcare systems worldwide, including in Indonesia, where efficient resource management is essential to improving patient outcomes and reducing the economic burden on both hospitals and patients [6,7].

The LOS for stroke patients is influenced by both unmodifiable and modifiable factors. Unmodifiable factors include stroke severity, affected brain region, and patient demographics (age, gender, and family history). Modifiable factors encompass conditions like hypertension, diabetes, heart disease, and cholesterol levels. A Malaysian study found that severe stroke patients tend to stay hospitalized longer than those with mild or moderate strokes [8]. In China, patients at high risk of stroke tend to have a longer hospital stay compared to those at lower risk [9], and on average, stroke patients remain hospitalized for more than two weeks, which is approximately twice the length of hospital stays in most Western countries [10]. However, these findings may not be directly applicable to Indonesia due to variations in stroke care accessibility, referral systems, and hospital capacity. Understanding how these factors play out in Indonesia is essential for developing targeted interventions to improve stroke care efficiency.

To bridge this gap, our research aims to identify the factors related with the LOS of ischemic stroke patients at National Brain Center Hospital Mahar Mardjono Jakarta (NBCH), the national referral center for stroke cases in Indonesia. This study seeks to enhance the broader understanding of medical cost management in stroke care, ultimately helping healthcare systems optimize their operations for better patient outcomes.

## Materials and methods

### Study design and outcomes

This research employed a retrospective cohort study, analyzing secondary data from the ischemic stroke patient registry at the NBCH. The data, covering the period from January 1 to December 31, 2020, was sourced from the hospital’s electronic medical records system, comprising 3,549 patient records.

The primary outcome was to identify factors related to LOS for ischemic stroke patients, while the secondary outcome was to examine LOS factors based on stroke severity.

### Ethical consideration

Prior to initiating the research, an ethics clearance application was submitted to the Health Ethics Committee of the National Research and Innovation Agency. The committee approved the application, as stated in letter number 117/KE.03/SK/11/2023. The research was approved to proceed without the need for informed consent from individual participants, as it relied solely on anonymized data. This data, provided by the hospital’s data team, was shared with us on April 4, 2024.

### Study participants

The study’s inclusion criteria were: all patients over 18 years old admitted to the hospital with a diagnosis of ischemic stroke during the specified period were included. Patients who refused hospitalization or had incomplete data were excluded from the analysis.

### Data collection

All data was obtained from the hospital’s electronic medical records system. The secondary data was collected by the stroke registry team under the supervision of the head of the stroke registry and was subsequently reviewed by the principal investigator. The LOS was defined as the time between the patient’s registration in the emergency department and their discharge from the ward, measured in days. Ischemic stroke is a neurological dysfunction caused by localized infarction in the brain, spinal cord, or retina [11]. Conservative treatment involves non-invasive management, like antiplatelet therapy. Interventional treatment actively restores blood flow by reopening blocked vessels, such as with thrombolysis or thrombectomy. The sociodemographic variables collected included gender, age, marital status, employment status, ward class, payment type, and Indonesian National Health Insurance (NHI) class. Clinical data collected included medical history (stroke, hypertension, cardiovascular disease [CVD], and diabetes mellitus [DM]), stroke severity classified using the National Institutes of Health Stroke Scale (NIHSS), with minor (0–5), moderate (6–15), and severe (>15) [12]. Blood pressure was classified based on the 2021 ACC/AHA Hypertension Guidelines into four categories: normal (<120/80 mmHg), elevated (120–129/ < 80 mmHg), stage 1 hypertension (130–139/80–89 mmHg), and stage 2 hypertension (≥140/ ≥ 90 mmHg) [13]. Additional data included diagnoses of DM and dyslipidemia during hospitalization, family history of stroke, smoking status, as well as any treatments received or complications experienced during the hospital stay. Patient history and physical examinations were conducted in the emergency department. LOS was analyzed as a continuous variable to preserve its full variability and ensure broader applicability across different regions, as hospital-defined thresholds for “long” or “short” stays may vary. All other variables were treated as categorical.

### Statistical analysis

Means and standard deviations (SD) were calculated and presented for continuous variables. For categorical variables, count and percentages were calculated and presented. Pearson’s Chi-square test was used to compare the distribution of categorical variables between subgroups of stroke severity, while analysis of variance (ANOVA) was used to compare the distribution of continuous variables. Univariable zero truncated negative binomial (ZTNB) regression was performed to assess each potential factor and multivariable was performed for all potential factors. The incidence rate ratio (IRR), exponential of the coefficient, and its 95% confidence interval (CI) from ZTNB regression were reported. We used ZTNB regression because for LOS study, usually there was no patient with zero-day LOS and had right skewed LOS distribution. *P*-value < 0.05 was considered as significant in each analysis. All analyses were conducted using R Statistical Software (v4.5.1; R Core Team 2025).

## Results

### Patients’ characteristics

**Fig 1:**
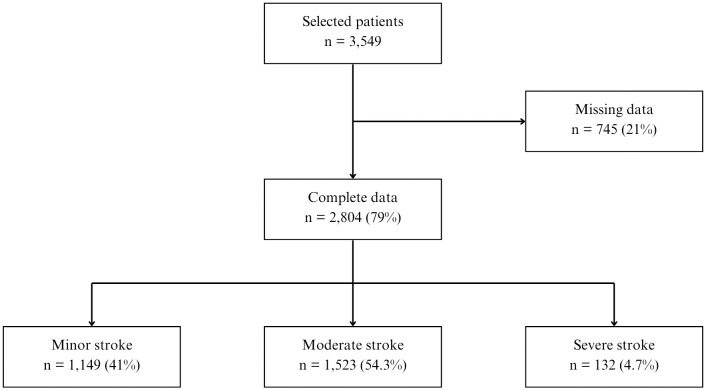
Patients’ data selection for analysis.

Out of 3,549 adult stroke ischemic admitted to NBCH in 2020, around fifth of them did not have complete data and must be excluded. From 2,804 patients with complete data, the majority was moderate stroke patients, followed by minor stroke, and only one of 20 patients had severe stroke (Fig 1). The mortality rate was very low (0.17%) as only 5 patients died during hospitalization. Means age of all patients was 61.3 years (SD ± 11.4) and around 6 out of 10 patients were male (63.5%). Only 13.2% of patients with not-married status and equal proportion between employed and unemployed patients. Almost half of patients (48.4%) were hospitalized in class 1 or VIP ward and just 5.8% paid the hospitalization cost without insurance, as seen in Table 2.

Around 3 out of 4 patients (72%) admitted to hospital had moderate or severe body mass index (BMI). Patients reported 23.8% previously had stroke, 88.2% had hypertension, 55.8% had DM, 27.7% had CVD, 19.5% were smokers, and 2% had close family members with stroke. Based on examinations at admission and during hospitalization, only 4.4% of patients had normal blood pressure, more than half (53.2%) had DM, and 81.8% had dyslipidemia. During the hospitalization, 6.1% of patients experienced complications and 6% of patients had to receive more than conservative treatment (Table 1).

**Table 1:** Characteristics of patients stratified by stroke severity. A) Demographics of patients stratified by stroke severity, B) Clinical characteristics of patients stratified by stroke severity, C) Interventions received during hospitalization stratified by stroke severity.

		Minor	Moderate	Severe	p-value
**Panel A**					
Total N		1,149	1,523	132	
Age (years)	Mean (SD)	**58.5 (10.8)**	**59.4 (10.6)**	**61.3 (11.4)**	**0.005**
Gender	Female	**381 (33.2)**	**586 (38.5)**	**57 (43.2)**	**0.005**
	Male	**768 (66.8)**	**937 (61.5)**	**75 (56.8)**	
Marital status	Married	**1024 (89.1)**	**1303 (85.6)**	**108 (81.8)**	**0.006**
	Not married	**125 (10.9)**	**220 (14.4)**	**24 (18.2)**	
Employment status	Employed	**619 (53.9)**	**725 (47.6)**	**52 (39.4)**	**<0.001**
	Unemployed	**530 (46.1)**	**798 (52.4)**	**80 (60.6)**	
Payment type	NHI	**982 (85.5)**	**1363 (89.5)**	**122 (92.4)**	**0.009**
	Private/co-share	**84 (7.3)**	**87 (5.7)**	**4 (3.0)**	
	OOP	**83 (7.2)**	**73 (4.8)**	**6 (4.5)**	
NHI class	1	**517 (45.0)**	**577 (37.9)**	**47 (35.6)**	**<0.001**
	2	**135 (11.7)**	**177 (11.6)**	**17 (12.9)**	
	3	**383 (33.3)**	**669 (43.9)**	**62 (47.0)**	
	Non-NHI	**114 (9.9)**	**100 (6.6)**	**6 (4.5)**	
**Panel B**					
Total N		1,149	1,523	132	
BMI	Under-normal	306 (26.6)	435 (28.6)	46 (34.8)	0.249
	Over	500 (43.5)	670 (44.0)	52 (39.4)	
	Obese	343 (29.9)	418 (27.4)	34 (25.8)	
Stroke history	Absent	**908 (79.0)**	**1151 (75.6)**	**78 (59.1)**	**<0.001**
	Present	**241 (21.0)**	**372 (24.4)**	**54 (40.9)**	
Hypertension history	Absent	151 (13.1)	166 (10.9)	14 (10.6)	0.187
	Present	998 (86.9)	1357 (89.1)	118 (89.4)	
Hypertension at admission	Normo	41 (3.6)	73 (4.8)	10 (7.6)	0.227
	Elevated	37 (3.2)	36 (2.4)	2 (1.5)	
	Stage1	376 (32.7)	483 (31.7)	41 (31.1)	
	Stage2	695 (60.5)	931 (61.1)	79 (59.8)	
DM history	Absent	669 (58.2)	827 (54.3)	68 (51.5)	0.078
	Present	480 (41.8)	696 (45.7)	64 (48.5)	
DM at admission	Absent	218 (19.0)	269 (17.7)	22 (16.7)	0.618
	Present	931 (81.0)	1254 (82.3)	110 (83.3)	
Dyslipidemia	Absent	**594 (51.7)**	**678 (44.5)**	**40 (30.3)**	**<0.001**
	Present	**555 (48.3)**	**845 (55.5)**	**92 (69.7)**	
CVD history	Absent	**890 (77.5)**	**1061 (69.7)**	**75 (56.8)**	**<0.001**
	Present	**259 (22.5)**	**462 (30.3)**	**57 (43.2)**	
Family history of stroke	Absent	1124 (97.8)	1498 (98.4)	127 (96.2)	0.185
	Present	25 (2.2)	25 (1.6)	5 (3.8)	
Smoking history	Absent	941 (81.9)	1214 (79.7)	102 (77.3)	0.234
	Present	208 (18.1)	309 (20.3)	30 (22.7)	
**Panel C**					
Total N		1,149	1,523	132	
Length of stay	Mean (SD)	**4.3 (2.3)**	**5.4 (3.5)**	**9.0 (5.8)**	**<0.001**
Ward class	1	**512 (44.6)**	**577 (37.9)**	**47 (35.6)**	**<0.001**
	2	**137 (11.9)**	**172 (11.3)**	**19 (14.4)**	
	3	**383 (33.3)**	**675 (44.3)**	**61 (46.2)**	
	VIP	**117 (10.2)**	**99 (6.5)**	**5 (3.8)**	
Hospital complication	Absent	**1125 (97.9)**	**1417 (93.0)**	**92 (69.7)**	**<0.001**
	Present	**24 (2.1)**	**106 (7.0)**	**40 (30.3)**	
Treatment	Conservative	**1101 (95.8)**	**1414 (92.8)**	**121 (91.7)**	**0.003**
	Intervention	**48 (4.2)**	**109 (7.2)**	**11 (8.3)**	

**Notes:** NHI = National health insurance, OOP = Out of pocket, BMI = Body mass index, DM = Diabetes mellitus, CVD = cardiovascular disease, VIP = Very important person. Comparison between subgroups of stroke severity for categorical variables was performed using Pearson’s chi-square and for continuous variables using analysis of variance.

Table 1A–1C showed the characteristics of the patients stratified by their stroke severity. Significant differences were found in patients’ sociodemographic characteristics, including age, gender, marital status, employment status, payment type, and NHI class (Table 1A). In terms of clinical characteristics, stroke history, dyslipidemia, and cardiovascular disease (CVD) history showed significant differences (Table 1B). When stratified by stroke severity, significant differences were also observed in ward class, hospital complications, and treatment type received during hospitalization (Table 1C).

**Fig 2:**
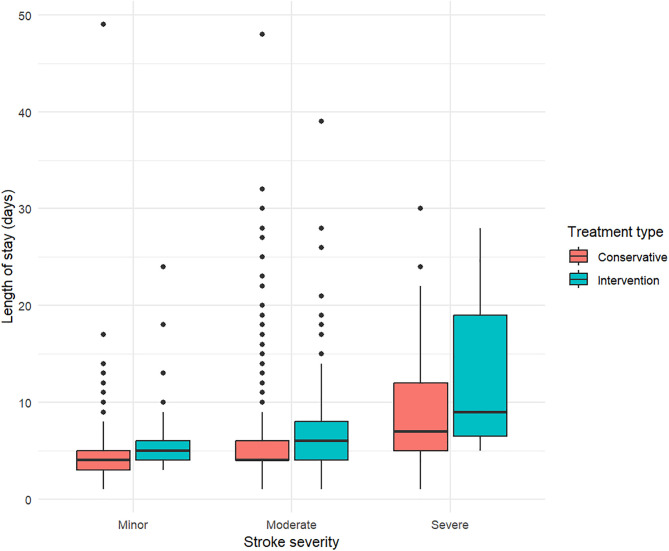
Box plot of length of stay stratified by stroke severity and treatment type.

Overall, the hospitalization duration was 5.1 days (SD ± 3.4). Patients with severe stroke had longer LOS, followed by patients with moderate stroke, and patients with minor stroke had shortest LOS (Fig 2). Further stratification by treatment type in Fig 2 showed that patients who had intervention treatment had longer LOS than the ones received conservative treatment. Although the maximum LOS in patients with minor and moderate stroke with conservative treatment was longer than the ones with intervention treatment. Fig 2 showed that there was no patient with zero day of LOS, and the distribution was skewed to the right, as indicated by a longer tail on the higher end of LOS values and the presence of several high LOS value outliers.

### Factors associated with length of stay in hospital

In univariable analysis for all patients, we found several factors were associated with LOS. Lower ward class and higher blood pressure were associated with shorter LOS, with 0.91 IRR (95% CI: 0.84 to 0.98) for class 2 ward, 0.74 IRR (95% CI: 0.62 to 0.87) for elevated blood pressure, 0.78 IRR (95% CI: 0.7 to 0.87) for stage 1 hypertension, and 0.82 IRR (95% CI: 0.74 to 0.9) for stage 2 hypertension. More severe stroke (1.27 IRR (95% CI: 1.21 to 1.33) for moderate and 2.17 IRR (95% CI: 1.99 to 2.36) for severe stroke), DM history (1.11 IRR (95% CI: 1.06 to 1.11)), DM at admission (1.22 IRR (95% CI: 1.16 to 1.27)), CVD history (1.37 IRR (95% CI: 1.31 to 1.43)), complication (2.4 IRR (95% CI: 2.25 to 2.56)) or received intervention treatment (1.51 IRR (95% CI: 1.39 to 1.64) days longer) during hospitalization were associated with longer LOS. Those variables showed similar results with slight change of effect size in multivariable analysis. However, some factors became significant or non-significant in the multivariable analysis. Private or co-share payment and out of pocket payment was associated with longer LOS (1.13 IRR (95% CI: 1.03 to 1.25) and 1.19 IRR (95% CI 1.09 to 1.3), respectively) while treated in VIP ward (0.87 IRR (95% CI 0.79 to 0.96)) and dyslipidemia (0.95 IRR (95% CI 0.9 to 0.999)) were associated with shorter LOS. Stage 2 hypertension and DM history were no longer significant in multivariable analysis (Table 2).

**Table 2:** Factors related to length of stay in hospital.

		Univariable		Multivariable	
		IRR (95% CI)	p value	IRR (95% CI)	p value
Age (years)	Mean (SD)	1.00 (1.00–1.00)	0.055	1.00 (1.00–1.00)	0.363
Gender	Female	–	–	–	–
	Male	0.97 (0.93–1.02)	0.252	0.98 (0.94–1.03)	0.461
Marital status	Married	–	–	–	–
	Not married	1.01 (0.95–1.08)	0.677	1.00 (0.94–1.06)	0.975
Employment status	Employed	–	–	–	–
	Unemployed	**1.06 (1.02–1.11)**	**0.009**	1.02 (0.97–1.07)	0.541
Ward class	1	–	–	–	–
	2	**0.91 (0.84–0.98)**	**0.009**	**0.91 (0.86–0.97)**	**0.006**
	3	0.96 (0.92–1.01)	0.134	0.97 (0.93–1.01)	0.137
	VIP	0.92 (0.84–1.00)	0.054	**0.87 (0.79–0.96)**	**0.004**
Payment type	NHI	–	–	–	–
	Private/co-share	1.03 (0.94–1.13)	0.489	**1.13 (1.03–1.25)**	**0.013**
	OOP	1.06 (0.97–1.17)	0.184	**1.19 (1.09–1.30)**	**<0.001**
Stroke severity	Minor	–	–	–	–
	Moderate	**1.27 (1.21–1.33)**	**<0.001**	**1.16 (1.11–1.21)**	**<0.001**
	Severe	**2.17 (1.99–2.36)**	**<0.001**	**1.56 (1.44–1.69)**	**<0.001**
BMI	Under-normal	–	–	–	–
	Over	1.00 (0.95–1.06)	0.880	1.01 (0.95–1.06)	0.832
	Obese	0.95 (0.90–1.00)	0.042	0.98 (0.93–1.03)	0.359
Stroke history	Absent	–	–	–	–
	Present	1.12 (1.07–1.18)	<0.001	1.02 (0.98–1.07)	0.392
Hypertension history	Absent	–	–	–	–
	Present	1.04 (0.97–1.11)	0.304	1.01 (0.94–1.07)	0.845
Hypertension at admission	Normo	–	–	–	–
	Elevated	**0.74 (0.62–0.87)**	**<0.001**	**0.85 (0.73–0.98)**	**0.026**
	Stage1	**0.78 (0.70–0.87)**	**<0.001**	**0.90 (0.82–0.99)**	**0.029**
	Stage2	**0.82 (0.74–0.90)**	**<0.001**	0.92 (0.84–1.01)	0.079
DM history	Absent	–	–	–	–
	Present	**1.11 (1.06–1.16)**	**<0.001**	1.01 (0.97–1.06)	0.601
DM at admission	Absent	–	–	–	–
	Present	**1.22 (1.16–1.27)**	**<0.001**	**1.09 (1.04–1.15)**	**<0.001**
Dyslipidemia	Absent	–	–	–	–
	Present	0.97 (0.91–1.02)	0.216	**0.95 (0.90–0.999)**	**0.038**
CVD history	Absent	–	–	–	–
	Present	**1.37 (1.31–1.43)**	**<0.001**	**1.24 (1.19–1.29)**	**<0.001**
Family history of stroke	Absent	–	–	–	–
	Present	1.05 (0.90–1.23)	0.545	1.05 (0.92–1.20)	0.451
Smoking history	Absent	–	–	–	–
	Present	1.05 (0.99–1.10)	0.111	0.97 (0.92–1.02)	0.186
Hospital complication	Absent	–	–	–	–
	Present	**2.40 (2.25–2.56)**	**<0.001**	**1.99 (1.87–2.12)**	**<0.001**
Treatment	Conservative	–	–	–	–
	Intervention	**1.51 (1.39–1.64)**	**<0.001**	**1.38 (1.29–1.48)**	**<0.001**

**Notes:** IRR = Incidence rate ratio, 95% CI = 95% confidence interval, VIP = Very important person, NHI = National health insurance, OOP = Out of pocket, BMI = Body mass index, DM = Diabetes mellitus, CVD = cardiovascular disease

The multivariable subgroup analysis showed multiple associated factors but only three factors were significantly associated in all groups (Table 3). Different ward class was significantly associated with shorter LOS in minor and moderate stroke groups. Higher BMI level associated with shorter LOS only in minor stroke group while above normal blood pressure and dyslipidemia only in moderate stroke group. Payment type and DM at admission were associated with longer LOS in minor and moderate stroke group. Finally, CVD history, hospital complication, and treatment type were associated with longer LOS in all stroke groups.

**Table 3:** Factors related to length of stay in hospital stratified by stroke severity.

		Minor		Moderate		Severe	
		IRR (95% CI)	p value	IRR (95% CI)	p value	IRR (95% CI)	p value
Age (years)	Mean (SD)	1.00 (1.00–1.00)	0.447	1.00 (1.00–1.00)	0.727	1.00 (0.99–1.01)	0.849
Gender	Female	-	-	-	-	-	-
	Male	0.97 (0.90–1.05)	0.499	0.97 (0.91–1.04)	0.445	1.10 (0.87–1.40)	0.429
Marital status	Married	-	-	-	-	-	-
	Not married	0.94 (0.85–1.03)	0.196	1.04 (0.97–1.12)	0.296	0.99 (0.76–1.28)	0.916
Employment status	Employed	-	-	-	-	-	-
	Unemployed	1.03 (0.96–1.11)	0.368	1.00 (0.94–1.07)	0.962	1.08 (0.84–1.39)	0.562
Ward class	1	-	-	-	-	-	-
	2	**0.88 (0.80–0.98)**	**0.015**	0.95 (0.87–1.04)	0.278	0.81 (0.60–1.10)	0.171
	3	0.98 (0.92–1.06)	0.663	0.95 (0.90–1.01)	0.088	1.07 (0.85–1.34)	0.573
	VIP	0.96 (0.84–1.09)	0.500	**0.81 (0.70–0.93)**	**0.003**	0.87 (0.47–1.60)	0.645
Payment type	NHI	-	-	-	-	-	-
	Private/co-share	1.09 (0.95–1.25)	0.238	**1.20 (1.05–1.38)**	**0.008**	0.80 (0.39–1.63)	0.537
	OOP	**1.13 (1.00–1.28)**	**0.044**	**1.21 (1.07–1.36)**	**0.002**	1.29 (0.79–2.09)	0.303
BMI	Under-normal	-	-	-	-	-	-
	Over	**0.91 (0.84–0.99)**	**0.024**	1.07 (1.00–1.15)	0.061	1.05 (0.83–1.33)	0.660
	Obese	**0.92 (0.86–0.99)**	**0.030**	1.03 (0.97–1.10)	0.372	0.88 (0.70–1.11)	0.277
Stroke history	Absent	-	-	-	-	-	-
	Present	1.05 (0.98–1.13)	0.158	1.00 (0.94–1.06)	0.915	1.09 (0.88–1.35)	0.440
Hypertension history	Absent	-	-	-	-	-	-
	Present	1.00 (0.91–1.10)	0.992	1.02 (0.94–1.11)	0.628	0.90 (0.65–1.25)	0.541
Hypertension at admission	Normo	-	-	-	-	-	-
	Elevated	0.97 (0.78–1.21)	0.799	**0.75 (0.61–0.92)**	**0.006**	1.34 (0.63–2.84)	0.453
	Stage1	0.95 (0.81–1.13)	0.575	0.89 (0.79–1.00)	0.054	0.94 (0.64–1.36)	0.728
	Stage2	1.01 (0.86–1.19)	0.912	**0.89 (0.79–1.00)**	**0.047**	0.97 (0.68–1.37)	0.859
DM history	Absent	-	-	-	-	-	-
	Present	0.94 (0.87–1.02)	0.124	1.04 (0.98–1.11)	0.221	1.00 (0.82–1.23)	0.988
DM at admission	Absent	-	-	-	-	-	-
	Present	**1.16 (1.08–1.25)**	**<0.001**	**1.07 (1.01–1.15)**	**0.034**	1.07 (0.84–1.36)	0.586
Dyslipidemia	Absent	-	-	-	-	-	-
	Present	1.03 (0.95–1.11)	0.459	**0.91 (0.85–0.97)**	**0.006**	1.02 (0.79–1.31)	0.901
CVD history	Absent	-	-	-	-	-	-
	Present	**1.15 (1.07–1.24)**	**<0.001**	**1.26 (1.19–1.34)**	**<0.001**	**1.46 (1.22–1.76)**	**<0.001**
Family history of stroke	Absent	-	-	-	-	-	-
	Present	1.07 (0.87–1.30)	0.530	1.03 (0.84–1.25)	0.789	1.13 (0.68–1.86)	0.645
Smoking history	Absent	-	-	-	-	-	-
	Present	0.94 (0.86–1.02)	0.138	1.00 (0.93–1.07)	0.992	0.86 (0.68–1.08)	0.185
Hosp complication	Absent	-	-	-	-	-	-
	Present	**2.35 (2.05–2.69)**	**<0.001**	**2.05 (1.89–2.22)**	**<0.001**	**1.64 (1.34–2.01)**	**<0.001**
Treatment	Conservative	-	-	-	-	-	-
	Intervention	**1.42 (1.25–1.61)**	**<0.001**	**1.34 (1.22–1.46)**	**<0.001**	**1.45 (1.06–1.97)**	**0.020**

**Notes:** IRR = Incidence rate ratio, 95% CI = 95% confidence interval, VIP = Very important person, NHI = National health insurance, OOP = Out of pocket, BMI = Body mass index, DM = Diabetes mellitus, CVD = cardiovascular disease.

## Discussion

Our retrospective study at Indonesia’s national stroke referral center provides novel insights into factors influencing the LOS for ischemic stroke patients, stratified by stroke severity. We based our analysis on findings by Ismail et al., who reported that greater stroke severity is associated with longer hospitalization [8]. Therefore, we aimed to validate these findings within the Indonesian population.

We found that shorter LOS was associated with Class II or VIP wards, while longer LOS was linked to out-of-pocket, private insurance, or cost-sharing payment methods. The reasons for these patterns remain uncertain; therefore, we considered ward class and payment type as proxies for socioeconomic status (SES) in the Indonesian healthcare context [14]. Patients with greater financial resources often choose higher ward classes that provide better amenities and privacy, whereas those with limited means tend to occupy lower ward classes. Likewise, payment method reflects SES, with wealthier patients more likely to use private insurance or cost-sharing, while lower-income patients typically rely on the NHI program.

Under NHI, stroke care is reimbursed through predetermined Indonesian Case-Based Group packages [15], which restrict the ability to extend hospitalization beyond what is covered. In contrast, patients paying out of pocket may be more inclined to prolong hospital stays to maximize recovery. A 2015 Indonesian report also noted that higher-income patients were slightly more likely to incur out-of-pocket expenses [16], a finding that contrasts with international studies. For example, research on 1.5 million stroke patients in the United States found that those with Medicaid or no insurance had longer hospital stays and poorer outcomes due to limited access to preventive care [17]. Another study showed that Medicare and Medicaid patients were more likely to remain hospitalized for two or more days [18].

Although we could not fully explain why Class II and VIP patients had shorter LOS while certain payment methods were linked to longer LOS, our findings underscore the significant role of socioeconomic factors in shaping hospitalization duration. The influence of SES, however, may vary according to patient characteristics and healthcare system structure.

Another factor influencing LOS is hypertension. Our study found that patients with elevated or stage I hypertension had shorter hospital stays, which contrasts with previous research. Hypertension is typically seen as a predictor of longer LOS, increasing treatment costs. For instance, Giani et al. found that in hypertensive emergencies, higher systolic blood pressure at presentation, especially in untreated patients, was linked to longer stays [19]. Similarly, other studies also associated hypertension with longer LOS [8,20–22]. However, several studies found no significant effect of hypertension on LOS [23,24]. Given these mixed findings, blood pressure patterns across ischemic stroke subtypes should be considered, as they directly impact patient outcomes and hospital stay duration. For example, normal blood pressure is more commonly observed in patients with cardioembolic stroke [25], whereas elevated blood pressure is frequently seen in those with atherothrombotic stroke [26]. Since cardioembolic strokes tend to be more severe than atherothrombotic strokes [27], they may lead to a longer hospital stay. However, due to conflicting findings in the existing literature, the lack of stroke subtype data in our study, and potential factors such as early discharge practices by some neurologists and patient selection bias, further research is needed to clarify the mechanisms behind this relationship.

We also observed that patients with dyslipidemia had shorter hospital stays compared to those without it. The link between dyslipidemia and LOS in ischemic stroke patients has yielded mixed results in various studies. For example, Indrayani et al. found a weak negative correlation between total cholesterol, LDL, and HDL levels and LOS, suggesting higher lipid levels might be associated with shorter stays [28]. However, another study found no significant relationship between lipid profiles at admission and hospital stay length in stroke patients [29].

In our study, a history of CVD and diabetes significantly extended hospital stays for ischemic stroke patients. Our findings on diabetes are consistent with previous research [30], suggesting that better glycemic control could help reduce LOS in future cases. However, studies found that a history of CVD did not significantly impact LOS [31,32], which contrasts with our results. Additionally, we found that complications and interventions, such as thrombolysis and thrombectomy during hospitalization, were linked to longer hospital stays, consistent with previous studies [33,34].

Lastly, stroke severity is a key factor in determining hospital LOS, with more severe strokes leading to longer stays. In our analysis, patients with severe strokes had an average LOS of 9 days, moderate strokes 5.4 days, and minor strokes 4.3 days. Studies consistently show that stroke severity, measured by the NIHSS, remains a strong and reliable predictor of both acute and total LOS [35,36], particularly in Asia countries [8,9]. Chang et al. found that for each 1-point increase in the NIHSS score, LOS increased by about 1 day for patients with mild to moderate strokes (NIHSS score 0–15). Interestingly, for patients with severe strokes (score >15), LOS decreased by 1 day [31], likely due to higher mortality rates in this group, leading to shorter stays. In cases of severe stroke, it is important to provide clear prognostic information and prepare for supported discharge services as early as possible, enabling families to make timely decisions and avoid unnecessary prolonged hospital stays.

This study highlights the importance of investing in cerebrovascular risk factors promotion and primary prevention to reduce the incidence of cardiovascular disease, which can help shorten hospital LOS and ease the financial burden of stroke. To support this, the Indonesian Ministry of Health introduced several health initiatives. In 2007, it launched the ‘CERDIK’ campaign, which stands for *Cek kesehatan* (regular health check-ups), *Enyahkan asap rokok* (eliminate cigarette smoke), *Rajin olahraga* (regular physical activity), *Diet seimbang* (balanced diet), *Istirahat cukup* (adequate rest), and *Kelola stres* (stress management) [37]. This was followed in 2016 by the ‘Gerakan Masyarakat Hidup Sehat’ (Healthy Lifestyle Movement), which encouraged the public to adopt healthier habits such as eating more fruits and vegetables, and reducing alcohol consumption [38]. Most recently, in early 2025, the Ministry introduced a new policy providing free annual medical check-ups for all age groups, tailored to age and health status, to improve risk factor management and prevent disease onset [39]. In addition, strengthening in-hospital stroke management pathways is essential to minimize post-stroke complications. Aligned with this need, in 2022 the Ministry of Health established the Indonesia Hospital Stroke Network under the National Stroke Care Transformation Program, aiming to create a more integrated stroke care system across the country [40].

Despite being conducted at Indonesia’s national referral center for stroke cases in a densely populated area of Jakarta, the capital city of Indonesia, this study has several limitations. About 21% of the data were excluded due to missing or incomplete information, including 167 deceased patients and 4 surviving patients with severely incomplete records, and 574 with missing education status. These exclusions, driven by the study’s retrospective design and reliance on secondary data, may introduce bias and affect the accuracy of our findings. As a single-center study, its applicability to the broader Indonesian population or national stroke care practices is limited. Additionally, our hospital’s policy capping ischemic stroke patient stays at five days may artificially restrict LOS, masking underlying factors influencing hospitalization duration. Furthermore, ischemic stroke is rarely classified by subtype in our setting, preventing subtype-based analysis. Lastly, data limitations may have excluded certain predictors from our regression analysis, potentially overlooking key factors affecting LOS. These limitations should be considered when interpreting the study results.

Future research should adopt a multi-center, prospective design to improve data completeness and generalizability across Indonesia. Categorizing ischemic stroke by subtypes will enable a more detailed analysis of its impact on LOS. Expanding the dataset to include socioeconomic factors, rehabilitation access, post-stroke complications, and genomic markers could provide a more comprehensive understanding of LOS determinants. Integrating predictive models using clinical and genomic data could enhance personalized stroke management. Additionally, assessing the impact of hospital policies, such as the 5-day stay cap, on patient outcomes may inform evidence-based care recommendations.

In summary, a history of CVD, hospital complications, and therapeutic interventions during hospitalization were significant predictors of LOS across all stroke severity levels. These findings emphasize the importance of investing in cerebrovascular risk factors promotion and primary prevention, along with early risk management and well-structured therapies, to enhance patient outcomes and hospital efficiency in Indonesia.

## Supporting information

S1 Data R script for the manuscript analysis. (R)
